# Free Mitochondrial DNA and Mitophagy Dysfunction in Diabetic Kidney Disease: A Central Mitochondrial–Inflammatory Axis Driving Progressive Renal Injury

**DOI:** 10.3390/cells15191746

**Published:** 2026-09-24

**Authors:** Aasthika Das, Eric Lalu, Vikky Awasthi, Cyrine Ben Dhaou, Hebaalaha Hussein, Glenn Gerhard, Karim Bahmed, Iris Lee, Vishwajeet Puri, Rihab Bouchareb

**Affiliations:** 1 Department of Translational Research, Temple University, Philadelphia, PA 19140, USA; 2 Department of Pathology and Translational Pathobiology, Louisiana State University Health Sciences Center-Shreveport, Shreveport, LA 71103, USA; 3 Center for Cardiovascular Research, Abigail Wexner Research Institute, Nationwide Children’s Hospital, Columbus, OH 43205, USA; 4 The Heart Center, Nationwide Children’s Hospital, Columbus, OH 43205, USA; 5 Center for Inflammation and Lung Research, Temple University, Philadelphia, PA 19140, USA; 6 Department of Nephrology, Hypertension & Kidney Transplantation, Lewis Katz School of Medicine, Temple University, Philadelphia, PA 19140, USA; 7 Diabetes Institute, Heritage College of Osteopathic Medicine, Ohio University, Athens, OH 45701, USA

**Keywords:** mitochondrial DNA, DKD, Kidney, inflammation

## Abstract

Diabetic kidney disease (DKD) remains the leading cause of chronic kidney disease and end-stage renal failure worldwide despite significant advances in glucose-lowering and renoprotective therapies. Emerging evidence identifies mitochondrial dysfunction as a central pathogenic mechanism linking metabolic stress to chronic inflammation and progressive renal injury. In the diabetic kidney, hyperglycemia, lipotoxicity, and oxidative stress impair mitochondrial quality control pathways, particularly mitophagy, a targeted mitochondrial degradation pathway, leading to the accumulation of dysfunctional mitochondria and increased mitochondrial reactive oxygen species production. A critical consequence of mitochondrial damage is the cytosolic release of mitochondrial DNA (mtDNA), which acts as a potent damage-associated molecular pattern and can activate innate immune pathways, including cGAS–STING, NLRP3 inflammasome, and Toll-like receptor 9 signaling. Persistent mtDNA-driven immune activation promotes sterile inflammation, cellular senescence, extracellular matrix remodeling, and renal fibrosis, thereby establishing a vicious cycle that accelerates DKD progression. In this review, we examine the emerging role of the mitochondrial DNA–inflammatory axis in DKD, highlighting the interplay between mitophagy dysfunction, mtDNA release, innate immune activation, and fibrotic remodeling. We further discuss the potential of circulating and urinary mtDNA as biomarkers of disease activity and evaluate therapeutic strategies targeting mitochondrial quality control, oxidative stress, and mtDNA-sensing pathways. Collectively, these findings position mtDNA as a critical mechanistic link between mitochondrial dysfunction and chronic inflammation, and support targeting the mitochondrial–immune axis as a promising strategy for precision therapies in DKD.

## 1. Introduction

Diabetic kidney disease (DKD) is the leading cause of chronic kidney disease (CKD) and end-stage renal disease worldwide and is one of the most serious complications of diabetes mellitus. Approximately 30–40% of patients with diabetes develop renal impairment and have an increased risk of cardiovascular morbidity and mortality [1,2].

DKD is characterized by glomerular hyperfiltration, albuminuria, mesangial expansion, podocyte injury, and progressive tubulointerstitial fibrosis [3]. Although strict glycemic control and reno-protective therapies such as renin–angiotensin–aldosterone system inhibitors (RAASis) and sodium-glucose cotransporter-2 (SGLT2) inhibitors have improved clinical outcomes, many patients still progress toward renal failure [4]. These observations highlight the need to better understand the molecular mechanisms driving DKD progression.

In recent years, mitochondrial dysfunction has emerged as a central contributor to diabetic kidney injury. The kidney is among the most energy-demanding organs in the body, and renal tubular epithelial cells rely heavily on mitochondrial oxidative phosphorylation (OXPHOS) to support active solute transport and metabolic homeostasis [5,6]. However, in the diabetic milieu, chronic hyperglycemia, lipid overload, and oxidative stress disrupt mitochondrial homeostasis, leading to impaired ATP production, excessive reactive oxygen species (ROS) generation, and structural abnormalities in mitochondrial networks [7]. These mitochondrial alterations contribute to metabolic reprogramming, inflammation, and the activation of profibrotic pathways, thereby promoting progressive renal injury.

A key mechanism that preserves mitochondrial integrity is mitophagy, a specialized form of autophagy that selectively removes damaged or dysfunctional mitochondria. Mitophagy is primarily regulated by the PTEN-induced kinase 1 (PINK1) and Parkin pathway, as well as receptor-mediated mechanisms involving proteins such as BNIP3, NIX, and FUNDC1 [8,9,10]. Under physiological conditions, mitophagy maintains mitochondrial quality control and prevents the accumulation of dysfunctional organelles that could compromise cellular homeostasis. However, increasing evidence indicates that mitophagy is impaired in DKD [11]. Studies in diabetic animal models and human kidney samples have demonstrated reduced mitophagy activity, mitochondrial fragmentation, and accumulation of damaged mitochondria in renal tubular cells and podocytes [11]. This impairment exacerbates oxidative stress and promotes cell injury, apoptosis, and fibrotic remodeling within the diabetic kidney.

One important consequence of mitochondrial damage is the release of mitochondrial DNA (mtDNA) into the cytosol and the extracellular environment. mtDNA retains several bacterial characteristics, including a circular structure and unmethylated CpG motifs, making it highly immunogenic when released from mitochondria [12,13]. As a result, mtDNA acts as a damage-associated molecular pattern (DAMP) that can activate innate immune pathways (Figure 1). Cytosolic mtDNA can trigger inflammatory signaling through multiple mechanisms, including activation of the cyclic GMP-AMP synthase (cGAS)-stimulator of interferon genes (STING) pathway, Toll-like receptor 9 (TLR9) signaling, and the NLRP3 inflammasome [14,15]. Activation of these pathways promotes the production of pro-inflammatory cytokines and amplifies ‘sterile inflammation’ (inflammation in the absence of viral or bacterial insults) [16] within injured tissues.

Free mtDNA released into the extracellular space is not the only immunostimulatory form of this DAMP. mtDNA can also be selectively packaged into extracellular vesicles (EVs), exosomes, and shed microvesicles carrying mitochondrial cargo, and exported in this vesicle-encapsulated form [17,18]. This distinction is functionally important: EV encapsulation protects mtDNA from extracellular nucleases, extends its half-life, and allows it to be trafficked to, and taken up by, cells well beyond the immediate site of injury [17]. Direct evidence that vesicle-contained mtDNA is sensed by cGAS–STING in the recipient cell now exists in several disease settings: tumor- and senescent-cell-derived EVs carrying mtDNA activate cGAS–STING–NF-κB signaling in recipient myeloid cells [19,20]; hepatocyte-derived, mtDNA-enriched EVs activate cGAS–STING signaling in skeletal muscle in cirrhosis [21]; and hypoxic tumor-derived EV-mtDNA activates macrophage cGAS/STING/NF-κB signaling [22]. In a stress paradigm closely analogous to the oxidative and metabolic stress of the diabetic kidney, sublethal oxidant exposure of epithelial cells triggers packaging and release of mtDNA into EVs together with activation of cGAS and NLRP3 signaling and markers of cellular senescence [18]. Whether renal-derived EV-mtDNA specifically engages cGAS–STING in DKD has not yet been directly tested and is, in our view, an important open question.

Emerging evidence suggests that mtDNA release and mitochondrial stress play critical roles in the pathogenesis of DKD. Experimental studies have shown that defective mitophagy and mitochondrial injury lead to cytosolic accumulation of mtDNA, activation of inflammatory signaling pathways, and promotion of renal fibrosis [23,24]. Moreover, elevated levels of circulating and urinary mtDNA have been detected in patients with diabetic nephropathy and correlate with disease severity [25,26]. These findings support the concept that mitochondrial dysfunction is not merely a consequence of diabetic injury but rather a key driver of inflammatory and fibrotic responses in the kidney.

Collectively, these observations suggest the existence of a mitochondrial–inflammatory axis in DKD. In this model, metabolic stress associated with diabetes disrupts mitochondrial quality-control mechanisms, such as mitophagy, leading to the accumulation of dysfunctional mitochondria and the release of mtDNA. Understanding the interplay between mitochondrial dysfunction, mitophagy impairment, and mtDNA-mediated inflammatory signaling may therefore provide new insight into the mechanisms underlying DKD progression.

In this review, we discuss the emerging role of mitophagy dysfunction and mitochondrial DNA (mtDNA) release in diabetic kidney disease (DKD). We first summarize the current knowledge of mitochondrial function and quality-control mechanisms in renal cells. We then examine the molecular pathways that regulate mitophagy and how these processes become dysregulated in the diabetic kidney. Finally, we explore how mtDNA acts as a danger-associated molecular pattern (DAMP) that activates innate immune and inflammatory pathways and discuss the therapeutic potential of targeting mitochondrial quality control to prevent or slow the progression of DKD. Although mitochondrial dysfunction in DKD has been reviewed previously, most prior reviews have considered mitochondrial bioenergetic failure, impaired mitophagy, and innate immune activation as largely distinct processes [27,28,29]. In contrast, this review integrates these interconnected mechanisms into a unified mitochondrial–inflammatory axis, with particular emphasis on cell-free mtDNA as a mechanistic link between upstream mitochondrial quality-control failure and downstream sterile inflammation and fibrosis. We further highlight the translational implications of this axis, including its potential for biomarker development and the identification of novel precision therapeutic strategies for DKD.

## 2. Mitochondrial Dysfunction in Diabetic Kidney Disease

Mitochondrial dysfunction is increasingly recognized as a central feature of DKD, linking chronic metabolic stress to renal injury, inflammation, and loss of kidney function [30,31,32,33,34,35,36]. Rather than a single abnormal state, mitochondrial alterations in DKD are dynamic and context-dependent, varying by disease stage, renal cell type, substrate availability, and local hemodynamic conditions. Some DKD cases may be characterized by increased substrate pressure and oxidative stress, whereas others show impaired oxidative metabolism, defective fatty acid oxidation, and bioenergetic insufficiency [7,32,33,34,37,38,39]. Thus, the hallmark of mitochondrial dysfunction in DKD is a reduced capacity to adapt to persistent diabetic stress.

It is important to emphasize that hyperglycemia is one of several convergent metabolic stressors, not the sole upstream trigger, and that correcting hyperglycemia alone would not be expected to fully reverse mitochondrial injury in DKD. Proximal tubular epithelial cells in particular rely preferentially on fatty-acid β-oxidation, rather than glycolysis, to meet their high ATP demand [39], and lipotoxicity from free fatty acid (FFA) and albumin-bound FFA overload directly impairs mitochondrial membrane potential, redox balance, and viability in proximal tubular cells independently of glucose concentration [40]. Genetic disruption of mitochondrial fatty-acid-linked ATP transport (ANT2 ablation) protects renal proximal tubules against obesity-induced CKD, even in the absence of diabetes, by forcing a shift away from fatty-acid oxidation [41], underscoring that lipid overload is an independently sufficient driver of tubular mitochondrial dysfunction rather than a minor cofactor of hyperglycemia. Oxidative stress, in turn, is generated by and amplifies both hyperglycemic and lipotoxic insults. We note that many of the specific molecular mechanisms described below (ROS-mediated mtDNA damage, PGC-1α suppression, DRP1/MFN2/OPA1 dysregulation) have been demonstrated predominantly in high-glucose-only in vitro systems that isolate hyperglycemia as a single experimental variable and do not capture the combined lipotoxic and oxidative burden present in vivo [7]; where a cited study used high glucose as the sole variable, or lipotoxic/combined stressors instead, we specify this below.

The severity of mitochondrial dysfunction in DKD differs across renal cell populations. Proximal tubular epithelial cells, which carry an especially high mitochondrial density to support solute reabsorption, are particularly vulnerable to bioenergetic failure and ROS-driven injury under hyperglycemic conditions. Podocytes, as terminally differentiated, non-regenerating cells, rely heavily on intact mitophagy for long-term survival, so even modest impairment of mitochondrial quality control can predispose these cells to irreversible loss and progressive proteinuria. Glomerular endothelial cells and mesangial cells appear comparatively less dependent on oxidative phosphorylation but remain susceptible to mtDNA-driven innate immune activation, contributing to microvascular injury and mesangial expansion [42]. This cell-type heterogeneity likely explains why mitochondrial-targeted interventions show variable efficacy across the different structural compartments of the diabetic kidney.

Hyperglycemia enhances mitochondrial substrate delivery, leading to increased electron transport chain (ETC) activity and excessive reactive oxygen species (ROS) production [43]. Mitochondrial ROS induces oxidative damage to lipids, proteins, and mtDNA. Reduced expression of PGC-1α, the master regulator of mitochondrial biogenesis, has been observed in diabetic kidneys and contributes to diminished mitochondrial renewal [44,45,46]. Altered mitochondrial dynamics (fission and fusion) further exacerbate dysfunction. Increased DRP1-mediated fission and reduced MFN2- and OPA1-mediated fusion promote fragmentation and membrane instability, increasing susceptibility to mtDNA leakage [47]. Beyond bioenergetic consequences, damaged mitochondria may release mtDNA into the cytosol or extracellular space, where it functions as a DAMP and activates innate immune pathways [24,48]. These findings underscore ROS-driven mtDNA injury as a mechanistic bridge between metabolic stress and renal damage [49].

Comparable ROS-driven mtDNA injury has been demonstrated with free-fatty-acid/lipotoxic stress alone, independent of hyperglycemia, in proximal tubular cells [40], indicating that this bridge is engaged by multiple convergent metabolic stressors rather than by hyperglycemia alone.

Although mitochondrial dysfunction and renal injury clearly reinforce one another once DKD is established, we take the position, central to the framework of this review, that mitochondrial dysfunction, and specifically defective mitochondrial quality control culminating in mtDNA release, acts as an initiating, upstream inducer of progressive DKD rather than merely a downstream bystander of glomerular and tubular injury. Three lines of evidence support this stance. First, mitochondrial abnormalities are detectable before overt renal injury: mitochondrial fragmentation and reduced PGC-1α expression precede albuminuria in early diabetic models [50], glomerular endothelial mitochondrial dysfunction is present before proteinuria and predicts susceptibility to nephropathy [51], and reduced renal oxidative metabolism is measurable in young adults with type 1 diabetes before clinically overt nephropathy [33]. Second, genetically manipulating mitochondrial quality-control genes alone, without any additional hyperglycemic insult, is sufficient to alter disease trajectory in either direction: tubule- or podocyte-specific deletion of fission/fusion or autophagy/mitophagy regulators worsens injury [35,36,52,53,54], whereas restoring these same pathways attenuates nephropathy [55,56,57]. Third, pharmacologically restoring mitochondrial quality control alone, without correcting glycemia, is sufficient to rescue the DKD phenotype [58,59]. Table 1 summarizes representative recent studies supporting this causal, upstream role. We therefore treat mitochondrial dysfunction as a driver of DKD throughout this review while recognizing that, once established, renal injury and its associated inflammatory and hemodynamic milieu subsequently feed back to further impair mitochondrial quality control, creating a self-reinforcing cycle rather than a simple linear cascade.

## 3. Mitophagy and Mitochondrial Quality Control

Mitophagy is a mitochondria-selective subtype of autophagy that additionally requires a distinct set of receptor and adaptor proteins, such as PINK1, Parkin, BNIP3, NIX/BNIP3L, FUNDC1, and OPTN, that specifically tag damaged mitochondria for engulfment [60]. In other words, every mitophagy event depends on the core autophagic machinery, but not every autophagic event involves mitophagy: impaired core autophagy (e.g., via mTORC1/TFEB dysregulation) and impaired mitophagy receptor signaling (e.g., PINK1/Parkin loss) are mechanistically distinct lesions that can occur together or independently in DKD. We treat them separately below and return to why this distinction matters for mtDNA release specifically in Section 4.

Mitophagy execution (PINK1–Parkin and receptor-mediated clearance). Mitophagy, a specialized form of macro-autophagy, is essential for maintaining mitochondrial integrity. Under physiological conditions, mitophagy selectively degrades damaged mitochondria, thereby preventing the persistence of dysfunctional organelles and limiting cellular stress [61]. In DKD, however, mitophagy is impaired, allowing damaged mitochondria to accumulate within renal cells [52,53,54,55,56]. A highly studied mitophagy pathway is the Parkin pathway (PINK1), which detects loss of mitochondrial membrane potential and promotes ubiquitination of outer membrane proteins, thus targeting damaged mitochondria for autophagic degradation [62,63]. This pathway is regulated by AMP-activated protein kinase (AMPK), mechanistic target of rapamycin complex 1 (mTORC1), and Transcription Factor EB (TFEB) (Figure 2).

Upstream autophagy regulation (AMPK–mTORC1–TFEB). AMPK is an important metabolic sensor that promotes the PINK1-Parkin pathway and is downregulated under hyperglycemia, resulting in reduced mitophagy [11]. mTORC1 is another nutrient-sensing signaling molecule that is activated by high glucose levels and suppresses autophagy [64]. Aberrant mTORC1 activation also phosphorylates TFEB, a master TF of autophagy and lysosomal-related genes, at serine 211. This phosphorylation inhibits TFEB’s nuclear translocation and further promotes impaired mitophagy [65]. Defective mitophagy results in the accumulation of dysfunctional mitochondria [66] (Figure 2). This persistence is not biologically neutral: dysfunctional mitochondria remain active sources of oxidative stress, membrane instability, and proinflammatory signaling, thereby sustaining tissue injury over time [52,53,54,55,56]. Experimental studies provide strong causal support for this concept. In diabetic mice, proximal tubule-specific autophagy deficiency exacerbates tubular injury, inflammation, fibrosis, and albuminuria, demonstrating that impaired organellar clearance can worsen renal disease progression [53]. Similarly, hyperglycemia-induced suppression of mitophagy has been linked to the accumulation of fragmented and depolarized mitochondria, increased ROS generation, and enhanced tubular apoptosis [52]. Disruption of core mitophagy regulators, including PINK1, further aggravates mitochondrial dysfunction, profibrotic signaling, and diabetic renal injury, whereas restoring mitophagy-related pathways improves mitochondrial respiration and attenuates DKD phenotypes in experimental models [54,55,56]. Together, these findings indicate that defective mitochondrial quality control converts an initially adaptive response to diabetic stress into a chronic self-reinforcing pathological state, a concept developed further below with respect to mitophagy’s temporal evolution and functional decline.

The severity of mitophagy impairment also appears to evolve over the course of disease. In early diabetes, mitophagy may be transiently upregulated as a compensatory response to increased mitochondrial damage, helping to preserve renal function. As hyperglycemic and oxidative stress persist, however, AMPK activity progressively declines, and mTORC1/TFEB signaling becomes more dysregulated, shifting mitophagy from an adaptive, protective response toward a chronically insufficient one. This temporal shift may help explain why interventions that enhance mitophagy tend to show the greatest benefit when introduced early in the disease course, before this compensatory capacity is exhausted.

In summary, the two arms of mitochondrial quality control described above are affected, and contribute to inflammation, in distinct ways in DKD. Impaired core autophagy signaling (AMPK–mTORC1–TFEB) reduces the cell’s overall lysosomal-degradative capacity, permitting the accumulation of misfolded proteins and additional damage-associated molecules alongside damaged mitochondria. Impaired mitophagy execution (PINK1–Parkin and receptor-mediated clearance) specifically spares damaged, mtDNA-replete mitochondria from degradation—the more direct route to the cGAS–STING, NLRP3, and TLR9 activation described in Section 5. Both arms nonetheless converge on renal inflammation, and both are described, together with their consequences for mtDNA handling, in Section 4.

## 4. Mechanisms of mtDNA Release

Maintaining mitochondrial health is essential for protecting renal function in diabetes [49]. Under diabetic conditions, hyperglycemia-driven overproduction of mitochondrial reactive oxygen species (ROS) can damage mitochondrial DNA (mtDNA) [49,67]. Additionally, hyperglycemia promotes the release of mtDNA into the extracellular space, where it can act as a DAMP and trigger inflammation [68,69]. One of the mechanisms of mtDNA release is through the mitochondrial permeability transition pore (mPTP), a stress-sensitive pore in the inner mitochondrial membrane (IMM) responsible for maintaining ionic balance by dissipating chemical/electrical ion buildup [70,71]. Under conditions of oxidative or metabolic stress, the mPTP can remain open for extended periods, resulting in mitochondrial swelling, outer membrane rupture, reduced (OXPHOS) due to loss of mitochondrial membrane potential, and release of mtDNA into the cytosol [70,72,73].

Mechanistically, mPTP-dependent release is closely tied to the quality-control failure described in Section 3: cyclophilin D-dependent pore opening is favored by the same mitochondrial Ca2+ overload and oxidative stress that accompany defective mitophagy, and once mtDNA reaches the cytosol through this route, it is a direct trigger for cGAS–STING activation, as shown for mPTP-dependent mtDNA release in kidney injury models [72] and for VDAC-dependent mtDNA export in diabetic podocytes [74].

Other mechanisms of mtDNA release include mitochondrial outer membrane permeabilization (MOMP), which involves BAX/BAK oligomers forming macropores on the outer membrane, resulting in herniation of the IMM and release of mtDNA into the cytoplasm [17].

In DKD specifically, BAX-mediated mtDNA leakage into the cytosol has been shown to directly activate cGAS–STING–TBK1/NF-κB signaling in podocytes independent of additional lipotoxic insult, and depleting cytosolic mtDNA, knocking down STING, or pharmacologically inhibiting STING or TBK1 each alleviates podocyte injury and improves renal function in diabetic and obese mouse models [75], providing direct mechanistic evidence that MOMP-mediated release, and not merely its downstream sensing, is causally linked to renal injury.

An indirect mode of mtDNA release occurs during deficient mitophagy [76]. Inefficient mitophagy causes only partial clearance of dysfunctional mitochondria, resulting in the cytosolic release of mtDNA [76]. Lysosomal dysfunction during mitophagy can also cause incomplete degradation of mitochondrial contents and contribute to cytosolic accumulation of mtDNA fragments [76].

Because PINK1–Parkin and receptor-mediated clearance normally removes whole damaged mitochondria, nucleoid and all, before they can rupture or be permeabilized, defective mitophagy increases the pool of mtDNA-replete mitochondria available to release their contents via the mPTP- and MOMP-dependent routes described above, in addition to allowing direct leakage of partially degraded mitochondrial contents from stalled autophagolysosomes [76].

### 4.1. Voltage-Dependent Anion Channel Oligomerization and Gasdermin-Mediated Release

Two additional mechanisms, mechanistically distinct from mPTP opening and BAX/BAK-dependent MOMP, are increasingly recognized in the broader mtDNA-inflammation literature and are directly relevant here. First, oxidatively stressed mitochondria can release short mtDNA fragments through pores formed by oligomerization of the voltage-dependent anion channel (VDAC) in the outer mitochondrial membrane, independent of BAX/BAK; positively charged residues in the VDAC1 N-terminal domain bind to mtDNA directly and promote this oligomerization, and pharmacological inhibition of VDAC oligomerization (VBIT-4) reduces mtDNA release and downstream interferon signaling in vivo [77]. VDAC1-dependent mtDNA export downstream of ceramide accumulation has already been demonstrated in diabetic podocytes specifically, directly linking this mechanism to DKD [74] (Section 4.2). Second, gasdermin D (GSDMD), the pore-forming executioner of pyroptosis, can translocate to and form pores in the mitochondrial membrane in addition to the plasma membrane; this GSDMD-mediated mitochondrial permeabilization causes mtDNA to leak into the cytosol, where it activates cGAS–STING and amplifies the same inflammatory cascade that produced active GSDMD in the first place, creating a self-amplifying feed-forward loop between pyroptosis and mtDNA-driven innate immune signaling [78,79]. Because NLRP3-inflammasome activation and caspase-1-dependent GSDMD cleavage are already implicated downstream of mtDNA sensing in DKD (Section 5), gasdermin-mediated mitochondrial permeabilization is a plausible amplification step linking the release mechanisms described here to the pyroptotic renal tubular injury reported in several DKD-specific studies (Table 2) [75,80,81]. These mechanisms are from different tissues; this specific feed-forward loop has not yet been directly demonstrated in renal cells and is presently an inference from other tissue contexts.

### 4.2. Vesicle-Mediated mtDNA Release

A fourth, mechanistically distinct route packages mtDNA, together with other mitochondrial components, into extracellular vesicles (EVs) for export, rather than releasing it directly into the cytosol. This occurs through at least two overlapping mechanisms: (i) selective sorting of damaged mitochondrial material into multivesicular bodies for exosomal release, which appears to serve partly as a protective mechanism that limits activation of cytosolic mtDNA sensors within the donor cell [86], and (ii) VDAC-dependent export of mtDNA fragments that are subsequently captured within microvesicles budding from the plasma membrane [19,74]. Both mechanisms have been demonstrated under metabolic and oxidative stress conditions directly relevant to DKD: sublethal oxidant exposure in epithelial cells triggers mitochondrial dynamic remodeling and mtDNA packaging into EVs alongside cGAS/NLRP3 pathway activation and markers of cellular senescence [18], and VDAC1-dependent mtDNA export downstream of ceramide accumulation drives cGAS–STING activation in diabetic podocytes [74]. Once released, EV-mtDNA can be trafficked to, and taken up by, resident or infiltrating immune cells and neighboring epithelial cells, propagating cGAS–STING activation beyond the original site of mitochondrial injury [19,20,21,22] (Section 1, Figure 1). Figure 3 summarizes all four mechanisms and their convergence on innate immune activation.

Mitochondrial DNA activates the immune system through three pathways: cGAS-STING, NLRP3 inflammasome, and TLR9. mtDNA is especially susceptible to oxidative damage due to its proximity to the electron transfer chain, its poor reparative capacity, and the lack of protection from histone-like proteins [87]. This oxidized mtDNA is highly inflammatory and promotes sterile inflammation, one of the hallmarks of DKD, via multiple pathways [16,88]. Oxidized mtDNA can activate the cyclic GMP-AMP (cGAS)–stimulator of interferon genes (STING) pathway, resulting in type I interferon responses [16]. mtDNA double-strand breaks (DSBs) also trigger BAX-BAK1-mediated mtRNA release into the cytosol, which activates the RIG-I-like receptor (RLR) signaling pathway, another inflammatory pathway that senses cytosolic RNA [89]. Furthermore, oxidized mtDNA can directly bind to the nucleotide-binding domain-like receptor (NLR) family pyrin domain-containing 3 (NLRP3) inflammasome, leading to the release of pro-inflammatory cytokines IL-1β and IL-18 [90].

Cyclic GMP-AMP synthase (cGAS) is a major intracellular pattern recognition receptor (PRR) in the innate immune system and is responsible for sensing cytosolic DNA. Once activated, cGAS synthesizes 2′-3′ cyclic GMP-AMP (cGAMP), which subsequently activates STING, or stimulator of interferon genes. Activated STING recruits and activates TANK-binding kinase (TBK1), which phosphorylates interferon regulatory factor 3 (IRF3). IRF3 then dimerizes and induces interferon production by inducing interferon-stimulated genes (ISGs) [91].

cGAS is the best-characterized cytosolic DNA sensor upstream of STING, but it is not the only one: the DEAD-box helicase DDX41 also binds to cytosolic DNA directly and signals through STING to activate TBK1–IRF3 and NF-κB, acting alongside and synergistically with cGAS in myeloid cells [92]. Whether DDX41 contributes to mtDNA sensing specifically in renal cells in DKD has not been examined and represents an unaddressed gap alongside the cGAS-centric model described above.

In DKD, the cGAS-STING pathway is chronically active because cGAS is activated by cytosolic mtDNA, resulting in long-term release of inflammatory cytokines that cause cellular senescence, in which cells permanently stop dividing but remain metabolically active, and fibrosis. Senescence is induced through STING-mediated downstream activation of nuclear factor kappa-light-chain-enhancer of activated B cells (NF-κB), leading to the secretion of senescence-associated secretory phenotype (SASP) (Figure 1), which is a mixture of interferons, matrix metalloproteases, and proteases [93]. The production of SASP promotes a self-perpetuating cycle that drives senescence and inflammation, and is potentially oncogenic due to T-cell death [94].

NOD-, LRR-, and pyrin domain-containing protein 3 (NLRP3) is a key intracellular sensor that activates the innate immune system and is known to promote inflammation in DKD [90]. Oxidized mtDNA (ox-mtDNA) has enhanced inflammatory potential, as it not only activates cGAS-STING but also directly binds to the NLRP3 inflammasome. Smaller fragments of ox-mtDNA bind to cytosolic NLRP3, which creates a complex with apoptosis-speck-like proteins, activating caspase-1, which in turn cleaves pro-IL-1β to pro-inflammatory IL-1β [95].

Toll-like receptor 9 (TLR9) is specialized to recognize unmethylated CpG islands in bacterial DNA and trigger an innate immune response [96]. Since mitochondria are evolutionarily derived from bacteria in an endosymbiotic event, mtDNA still contains bacterial motifs like unmethylated CpG islands and therefore activates TLR9 when free in the cytosol or extracellular space [97]. Once activated, TLR9 in turn activates NF-κB, leading to a proinflammatory response [96].

It is important to note that these three pathways are not mutually exclusive and can amplify and activate one another. For example, the cGAS-STING pathway, once activated by mtDNA, can concurrently activate the NF-κB pathway, amplifying the innate immune response [85]. Studies also suggest that the NLRP3 inflammasome is downstream of STING activation, and STING can recruit and deubiquitinate NLRP3, thereby activating the NLRP3 inflammasome [98].

## 5. Fibrosis and Cellular Senescence

Cellular senescence is a stable, largely irreversible state of cell-cycle arrest, mediated chiefly by p16^INK4a^ and p21^CIP1^, in which cells remain viable and metabolically active but stop proliferating and adopt a pro-inflammatory, pro-fibrotic senescence-associated secretory phenotype (SASP) [99]. Unlike programmed cell death, senescent cells persist within the tissue and continue to signal to their environment, which is what makes their accumulation pathogenic in chronic disease. Mitochondrial dysfunction, specifically mitophagy failure, is one of the primary drivers of this state: loss of mitochondrial quality control is considered a hallmark of the senescence program itself, and mtDNA released from damaged, non-mitophagized mitochondria, whether free or vesicle-packaged, is sufficient to trigger cGAS–STING-dependent senescence induction [20]. In DKD, this mitophagy–senescence relationship is direct, hyperglycemia-driven downregulation of PINK1, Parkin, and OPTN allows damaged mitochondria to accumulate in proximal tubular cells and podocytes, and this accumulation, rather than senescence arising as an independent, parallel process, is a principal trigger for cell-cycle arrest and SASP acquisition in these cells, as detailed below [100,101,102].

Cellular senescence is a physiological process involved in wound repair; however, in DKD, it can drive pathogenic tissue fibrosis [99]. Kidney cells, especially proximal tubular epithelial cells (PTECs), respond to insults such as oxidative and metabolic stress in a myriad of ways, including partial epithelial–mesenchymal transition (pEMT), metabolic reprogramming, cell cycle arrest, and cellular senescence. The common result of all these changes is the kidney cells becoming secretory and producing inflammatory and pro-fibrotic factors (SASP), which drive maladaptive fibrosis in DKD [103]. Mitochondrial dysfunction, such as impaired mitophagy due to hyperglycemia in DKD, can further induce senescence. High glucose levels downregulate mitophagic proteins such as PINK1, Parkin, and OPTN, resulting in the accumulation of damaged mitochondria and upregulating senescence [100,101]. In podocytes, increased mitochondrial fission promotes cell senescence. Specifically, deficiency of G-protein-coupled receptor 124 (GPR124) in DKD podocytes activates FAK, a component of integrin signaling, which increases mitochondrial DRP1 levels and upregulates mitochondrial fission, which again drives senescence [102].

Further, chronic inflammation in DKD due to a host of factors drives fibrosis, extracellular matrix (ECM) deposition, and scarring of renal tissue [104,105]. At its core, DKD is a product of hyperglycemia. For instance, the high glucose environment of the diabetic kidney promotes increased glucose reabsorption by the proximal tubular epithelial cells (PTECs), which requires increased ATP and places pressure on the mitochondria, resulting in increased ROS production as a byproduct of upregulated OXPHOS [66].

A clarification is warranted here: SGLT2-mediated glucose reabsorption itself is a secondary active transport process driven by the Na+ electrochemical gradient, not by direct ATP hydrolysis. The additional ATP demand under hyperglycemia arises predominantly from the basolateral Na+/K+-ATPase, which must continuously restore this Na+ gradient as glucose (and Na+) reabsorption increases; it is this downstream Na+/K+-ATPase workload, rather than SGLT2 transport per se, that places the additional bioenergetic burden on proximal tubular mitochondria [5,6].

Sustained hyperglycemia also promotes the production of pro-inflammatory cytokines, chemokines, adhesion molecules, toxic metabolites like advanced glycation end products (AGEs), and endoplasmic reticulum stress [66]. Simultaneously, pro-fibrotic signaling pathways like TGF-β1/Smad and Hippo-YAP/TAZ promote epithelial–mesenchymal transition (EMT) and extracellular matrix remodeling, all of which represent the pathogenic milieu of DKD [104,105]. Aberrant activation of the cGAS/STING/TBK1/p65 pathway also contributes to podocyte injury and maladaptive repair [106,107]. These processes collectively promote progressive interstitial fibrosis and a decline in renal function.

Mitophagy failure leading to accumulation of mtDNA-replete mitochondria is directly demonstrated within DKD models [11,52,53,54,55,56], as is mtDNA-driven cGAS–STING/NLRP3 activation in renal cells (Table 2). The link from cGAS–STING activation specifically to senescence induction, however, is substantiated mainly in non-renal systems (tumor and immune cells) [20], and its direct demonstration within diabetic renal tubular or podocyte cells remains inferential, reasoned by analogy rather than shown end-to-end in one experimental system. Similarly, the connection from renal cell senescence to fibrosis is well supported [103,108], but the specific sub-link between mitophagy-driven senescence (as opposed to senescence induced by other stressors) and fibrosis rests on a smaller set of DKD-specific studies [100,101,102] than the other steps in the chain. We flag this explicitly so that the overall model is read as a mechanistically plausible, partially validated framework rather than a fully proven, end-to-end causal pathway.

## 6. Biomarker Potential of mtDNA

The traditional biomarkers of DKD are albuminuria and declining eGFR; however, both fail to detect the early stages of the disease because they do not reflect mitochondrial dysfunction, and a substantial proportion of patients with diabetes have reduced eGFR in the absence of albuminuria, highlighting a need for markers that capture disease activity earlier [109].

Because damaged mitochondria release mtDNA into the extracellular space, several cross-sectional studies have reported that urinary and plasma mtDNA levels are elevated in patients with diabetic nephropathy and are associated with albuminuria and lower eGFR measured at a single time point [109,110], and mtDNA-driven inflammation is, in principle, an upstream event that could precede glomerular and tubular injury [110]. It is important to emphasize, however, that a cross-sectional association with disease severity is not evidence of prognostic or predictive value, and the two should not be conflated.

This distinction is illustrated directly by the available longitudinal data: in patients with biopsy-proven DKD followed for 24 months, elevated urinary mtDNA did not significantly predict subsequent renal function decline [26], a gap that may partly reflect the advanced, nephrotic-range proteinuria of that cohort, in whom proteinuria itself is already a strong independent predictor of progression, leaving little additional prognostic information for mtDNA to contribute. Testing mtDNA earlier in the disease course, before overt proteinuria, is a scientifically reasonable hypothesis, but any such study would need to be very large and long-running to be adequately powered, since most patients with normo- or microalbuminuria do not progress to advanced DKD; the cost and duration this would require are substantial, and we do not wish to overstate the near-term feasibility of this approach. Combined with the lack of methodological standardization across studies in specimen type, extraction method, normalization, target gene, and quantification platform, which limits cross-study comparison and precludes defined diagnostic or prognostic thresholds [111,112], we regard circulating and urinary mtDNA at present chiefly as a mechanistic biomarker that supports the mitochondrial–inflammatory axis described throughout this review, rather than as a likely biomarker, in its current form, to achieve regulatory-grade prognostic or predictive qualification for DKD.

## 7. Therapeutic Implications

Building on the mitochondrial–inflammatory mechanisms outlined above, therapeutic strategies are increasingly shifting towards targeting the mitochondrial–inflammatory axis rather than downstream injury alone [48,53,55,56,113,114,115,116]. Enhancing mitochondrial quality control, particularly mitophagy, is a key upstream approach, as diabetic conditions suppress AMPK and activate mTOR, impairing autophagic flux and allowing accumulation of ROS-producing dysfunctional mitochondria [53,114,115]. Pharmacologic activation of AMPK (e.g., metformin, AICAR) restores PINK1/Parkin-mediated mitophagy, improves mitochondrial biogenesis, and reduces fibrosis and albuminuria in experimental models [113,114], while mTOR inhibition restores protective autophagy [115]. Additional pathways, including ESRRA–ATG5 and TOMM7–PINK1/Parkin signaling, further support mitophagy restoration as a strategy to improve mitochondrial respiration and attenuate DKD progression [55,56]. Complementary approaches target mitochondrial oxidative stress using mitochondria-specific antioxidants such as MitoQ, SS-31, and SkQ1, which reduce mtROS, preserve mitochondrial structure and function, and suppress inflammasome activation and fibrosis [117,118,119,120,121,122]. These interventions not only improve mitochondrial bioenergetics but also limit mtDNA oxidation and release, thereby reducing downstream inflammatory signaling.

Targeting downstream mtDNA-sensing pathways provides an additional therapeutic axis, particularly in established disease. Cytosolic mtDNA activates cGAS–STING signaling and the NLRP3 inflammasome, driving chronic inflammation, fibrosis, and renal dysfunction [24,48,80,123,124,125,126,127,128]. Experimental inhibition of these pathways, via genetic deletion or pharmacologic agents such as RU.521 (cGAS inhibitor) or C176 (STING inhibitor), reduces inflammation and fibrosis in kidney injury models [24,48,80,123,124]. NLRP3 inhibition similarly improves renal outcomes across multiple disease contexts [125,126,127]. Importantly, combination strategies targeting multiple nodes of the mitochondrial–inflammatory axis show synergistic benefit, as demonstrated by dual modulation of mitophagy and inflammatory signaling [129,130]. However, translation remains challenging due to the context-dependent nature of mitochondrial signaling, variability across disease stages and patient populations, and the need for precise therapeutic targeting [24,25,31,55,56,74,80,131,132]. The development of biomarkers, such as urinary metabolomic signatures, mtDNA levels, and mitophagy-related gene expression, offers promise for patient stratification and monitoring [25,31,74,80,131]. Overall, integrated therapies targeting mitochondrial quality control, oxidative stress, and innate immune activation represent a next-generation, mechanism-based approach to slowing DKD progression and enabling precision medicine. Table 3 summarizes recent agents and strategies acting on this axis; next-generation approaches under active development include tissue-specific nanoparticle delivery of STING inhibitors, PROTAC-based STING degraders, and combination regimens pairing cGAS–STING or NLRP3 blockade with SGLT2 inhibitors or senolytics [133].

## 8. Conclusions

Cell-free mitochondrial DNA is a critical mechanistic link between metabolic stress and sterile inflammation in diabetic kidney disease. Mitophagy dysfunction amplifies mtDNA release, establishing a feed-forward loop of mitochondrial damage and immune activation. Targeting mitochondrial quality control and innate immune signaling pathways may offer novel therapeutic avenues to halt DKD progression.

Looking ahead, single-cell and spatial transcriptomic and multi-omic approaches are likely to be particularly valuable for advancing this field. Single-cell RNA sequencing can resolve the cell-type-specific patterns of mitochondrial dysfunction and mitophagy impairment described above, while spatial transcriptomics and proteomics can map mtDNA release and innate immune activation to specific anatomical niches within the diabetic kidney [135,136]. Integrating these datasets with circulating and urinary mtDNA measurements may ultimately allow the mitochondrial–inflammatory axis to be resolved at single-cell resolution in situ, refining biomarker strategies and identifying the specific cell populations and disease stages most likely to benefit from mitochondria-targeted therapies.

## Figures and Tables

**Figure 1:**
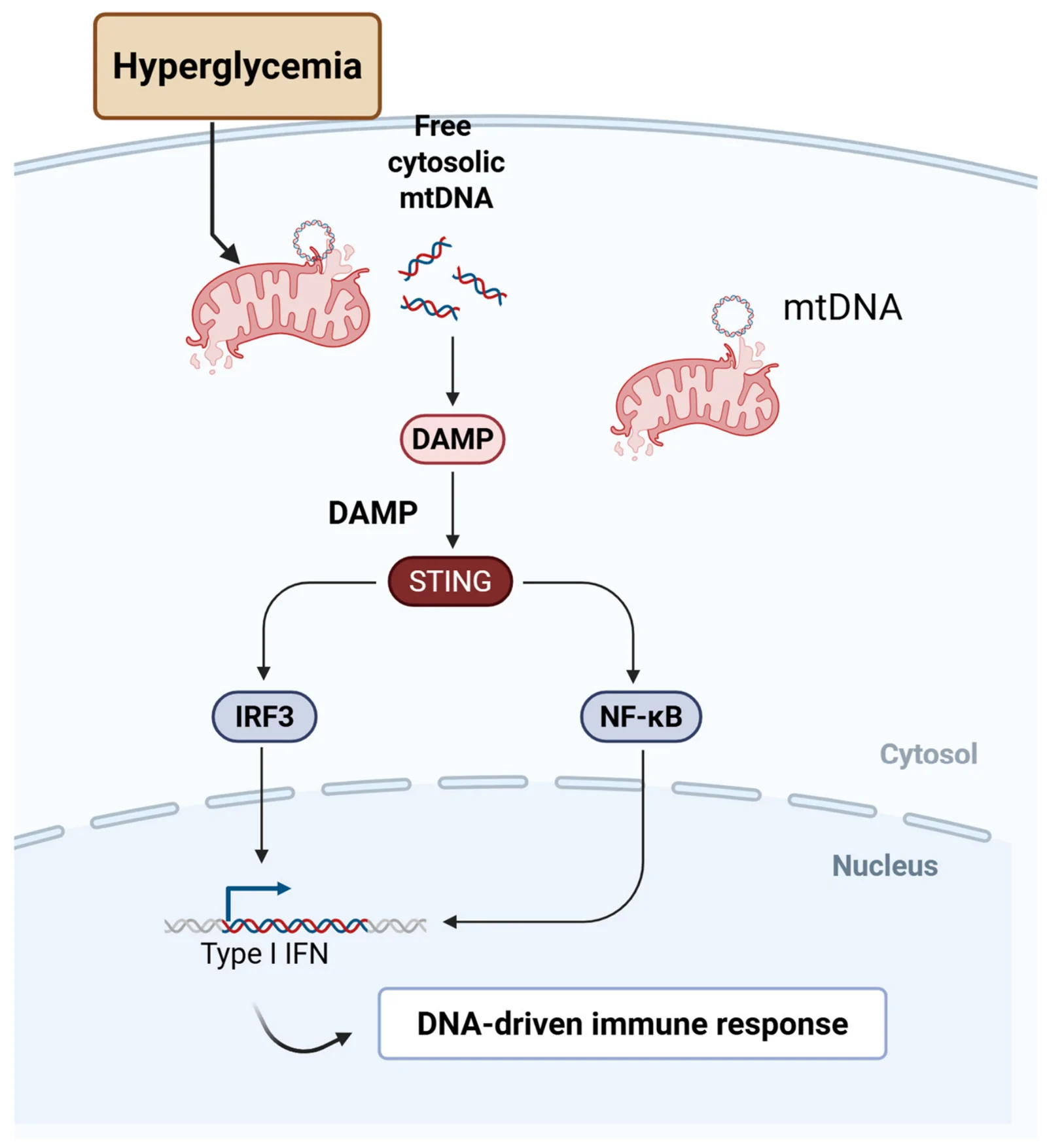
Mitochondrial DNA release activates sterile inflammation downstream of hyperglycemia, lipotoxicity, and oxidative stress. Mitochondrial injury induced by cellular stress compromises mitochondrial membrane integrity, resulting in the release of mitochondrial DNA (mtDNA) both as free/naked DNA and packaged within extracellular vesicles, into the cytosol and extracellular space. In either form, mtDNA functions as a potent damage-associated molecular pattern (DAMP). Cytosolic or vesicle-delivered mtDNA activates multiple innate immune pathways, including the cGAS–STING axis, leading to type I interferon signaling and NF-κB-mediated inflammatory responses. Collectively, these signaling pathways amplify the production of pro-inflammatory cytokines and chemokines, driving sterile inflammation and contributing to tissue injury, fibrosis, and disease progression in chronic disorders such as diabetic kidney disease.

**Figure 2:**
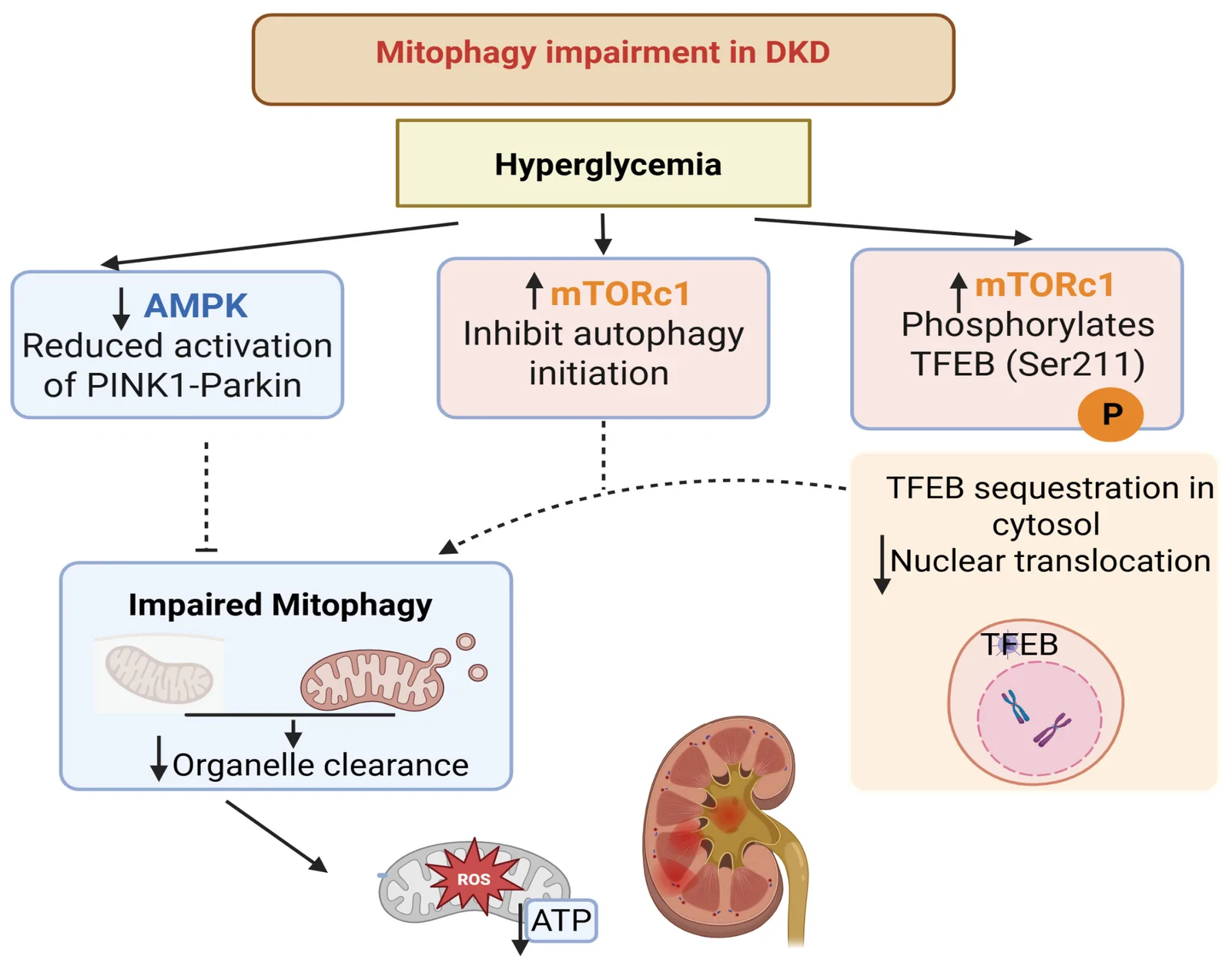
Upstream AMPK–mTORC1–TFEB autophagy signaling regulates PINK1–Parkin-mediated mitophagy in diabetic kidney disease. Under physiological conditions, damaged mitochondria are selectively removed through mitophagy, a mitochondria-selective subtype of autophagy, preserving mitochondrial quality, cellular bioenergetics, and redox homeostasis. AMPK and mTORC1/TFEB are core, non-mitophagy-specific regulators of autophagic flux and lysosomal biogenesis: AMPK promotes autophagy broadly and thereby supports PINK1–Parkin-mediated mitophagy, whereas mTORC1 suppresses autophagy under nutrient-rich conditions. In diabetic kidney disease, chronic hyperglycemia and lipotoxic free-fatty-acid excess both inhibit AMPK while persistently activating mTORC1. Activated mTORC1 phosphorylates TFEB at serine 211, preventing its nuclear translocation and reducing the transcription of autophagy- and lysosome-related genes. This upstream autophagy defect, in turn, impairs downstream PINK1–Parkin-mediated mitophagy specifically, leading to the accumulation of dysfunctional, mtDNA-replete mitochondria, increased oxidative stress, defective ATP production, and sustained renal inflammation and injury, thereby contributing to the progression of DKD.

**Figure 3:**
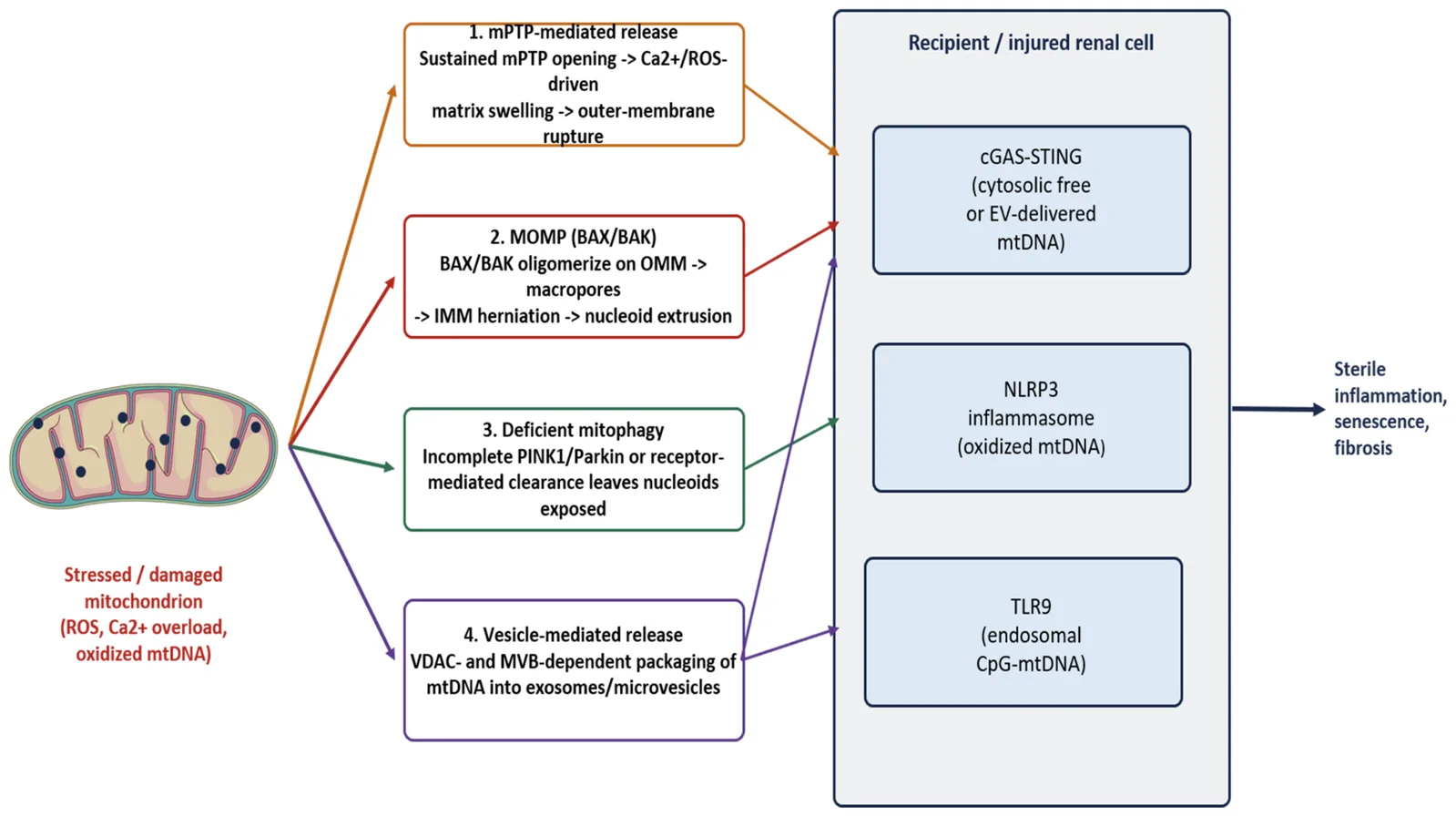
Multiple, non-mutually exclusive mechanisms release mitochondrial DNA into the cytosol and extracellular space in diabetic kidney disease. (1) Sustained opening of the mitochondrial permeability transition pore (mPTP) causes matrix swelling and outer-membrane rupture. (2) BAX/BAK oligomerization causes mitochondrial outer membrane permeabilization (MOMP) and macropore-mediated herniation of mtDNA nucleoids. (3) Deficient or incomplete mitophagy leaves damaged, mtDNA-replete mitochondria uncleared, permitting nucleoid escape. (4) mtDNA is selectively packaged into extracellular vesicles (EVs) via VDAC-dependent and multivesicular-body-dependent routes and exported to neighboring or distant cells. (5) Mitochondrial DNA-mediated inflammatory signaling in diabetic kidney disease.

**Table 1:** Representative recent evidence supporting mitochondrial dysfunction as an inducer of CKD/DKD progression. Studies are grouped to show convergent evidence from human, genetic loss/gain-of-function, and pharmacologic sources.

Study	Model/Design	Type of Evidence	Key Finding	Ref.
Choi et al., 2024	Young adults with type 1 diabetes	Human, early-stage	Attenuated renal oxidative metabolism detectable before overt nephropathy	[33]
Galvan et al., 2019	Drp1-S600 phospho-site knock-in diabetic mice	Genetic (loss of function)	Blocking fission-promoting DRP1 phosphorylation halts progression of nephropathy	[35]
Ayanga et al., 2016	Podocyte-specific Drp1-knockout diabetic mice	Genetic (loss of function)	Drp1 deletion improves mitochondrial fitness and prevents progression of diabetic nephropathy	[36]
Zhan et al., 2015	Renal tubular MIOX-overexpressing diabetic mice	Genetic/mechanistic	MIOX-driven disruption of mitochondrial quality control drives tubular injury	[52]
Ma et al., 2020	Proximal tubule-specific p53/miR-214/ULK1-axis autophagy-deficient diabetic mice	Genetic (loss of function)	Tubular autophagy deficiency alone worsens fibrosis, inflammation, and albuminuria	[53]
Sung et al., 2023	PINK1-knockout diabetic mice	Genetic (loss of function)	PINK1 loss aggravates mitochondrial dysfunction and necroptosis, worsening DKD	[54]
Hu et al., 2026; Wang et al., 2026	ESRRA–ATG5 and TOMM7–PINK1/Parkin gain-of-function diabetic models	Genetic/mechanistic (gain of function)	Enhancing mitophagy pathways alone improves mitochondrial respiration and attenuates DKD	[55,56]
Jiang et al., 2026	HK-2 cells and diabetic mice; pharmacologic mitophagy restoration (sEH inhibition)	Pharmacologic	Restoring mitophagy, independent of glycemic control, suppresses mtROS–NLRP3 inflammation and renal dysfunction	[57]
Mercurio et al., 2026	db/db mice, dietary intervention (Mediterranean-diet mix)	Pharmacologic/dietary	Preserving mitochondrial dynamics and autophagy/mitophagy balance alone attenuates early diabetic kidney injury	[58]
Yu et al., 2026	HK-2 cells and diabetic mice; targeted nanoparticle delivery of ginsenoside Rg1	Pharmacologic	Restoring mitochondrial homeostasis via AMPK/mTOR alone improves renal histopathology	[59]
Permyakova et al., 2024	Proximal tubule-specific ANT2-knockout obese mice (non-diabetic)	Genetic (loss of function)	Disrupting fatty-acid-linked mitochondrial ATP transport alone protects against obesity-induced CKD, independent of glycemia	[41]

**Table 2:** Recent original evidence for mtDNA-driven cGAS/STING activation in diabetic kidney disease. All studies below use DKD-relevant models (diabetic/obese mice or high-glucose-exposed human renal cells) and directly manipulate the mtDNA–cGAS–STING axis.

Study	Model	mtDNA/STING Mechanism	Key Finding	Ref.
Zang et al., 2022	Podocytes; diabetic and obese mice	BAX-mediated mtDNA leakage → cGAS–STING–TBK1/NF-κB	STING knockdown or pharmacologic inhibition (STING/TBK1) alleviates podocyte injury and improves renal function	[75]
Myakala et al., 2023	db/db diabetic mice	SIRT3-dependent mtDNA damage → cGAS–STING	Whole-body STING deletion or STING antagonism is renoprotective, independent of glycemia	[82]
Feng et al., 2023	HFD/STZ mice; HK-2 cells	PCSK9-driven mtDNA damage → cGAS–STING–NF-κB	PCSK9 blockade or STING inhibitor (C-176) reduces inflammatory cytokines and kidney injury	[83]
Zhu et al., 2025	Podocytes; diabetic mice; human DKD biopsies	CerS6-ceramide-VDAC1-mediated mtDNA leakage → cGAS–STING	Podocyte CerS6 knockout ameliorates glomerular injury; CERS6 correlates with proteinuria/GFR in human DKD	[74]
Huang et al., 2025	Renal tubular epithelial cells; diabetic mice	mtDNA–cGAS–STING → ISG15 → NLRP3–caspase-1–GSDMD pyroptosis	ISG15 knockout reduces fibrosis, inflammation, and pyroptosis	[80]
Guo et al., 2025	KK-Ay mice; HK-2 cells	High glucose/palmitate-induced mtDNA leakage → cGAS/STING	Direct mtDNA-transfection experiments confirm pathway specificity; herbal formula suppresses activation and renal injury	[84]
Li et al., 2026	STZ/nephrectomy rat model; HK-2 cells	AGE–Piezo1–Ca2+ → cGAS–STING → pyroptosis	Piezo1 knockdown or STING inhibitor (H-151) suppresses pathway and tubular pyroptosis	[81]
Yun et al., 2026	Review synthesis	TFAM stabilization prevents mtDNA leakage and cGAS–STING activation	Positions the TFAM–mtDNA–cGAS–STING axis as a mechanistic hub in DKD	[85]

**Table 3:** Recent therapeutic strategies targeting mtDNA release or cGAS/STING signaling in diabetic kidney disease and closely related renal fibrosis models.

Agent/Strategy	Target/Mechanism	Model	Outcome	Ref.
Nicotinamide riboside (NAD+ precursor)	Boosts SIRT3 activity, reducing mtDNA damage upstream of cGAS–STING	db/db diabetic mice	Reduced albuminuria and KIM-1; benefit comparable to STING antagonism/knockout	[82]
PCSK9 monoclonal antibody/siRNA	Reduces mtDNA damage and cGAS–STING activation	HFD/STZ mice; HK-2 cells	Reduced BUN, creatinine, UACR, and inflammatory cytokines	[83]
STING inhibitor C-176	Direct STING inhibition	HGPA-induced HK-2 cells (DN model)	Blocks the downstream inflammatory cascade	[83]
Qihuang Yishen formula (herbal)	Reduces mtDNA leakage; suppresses cGAS/STING	KK-Ay mice; HK-2 cells	Reduced proteinuria, tubular injury markers, and renal histopathological damage	[84]
M-SYFSF formula + STING inhibitor H-151	Piezo1–Ca2+–cGAS–STING–pyroptosis axis	STZ/nephrectomy DKD rats; HK-2 cells	Improved renal function; reduced fibrosis and tubular pyroptosis	[81]
Acacetin (flavonoid)	Direct STING binding/inhibition; rebalances mitophagy vs. protective “nucleoid-phagy”	UUO and folic-acid renal fibrosis mice (non-diabetic; mechanistically transferable)	STING-dependent reduction in fibrosis and oxidative stress	[134]
sEH inhibition	Restores PINK1/Parkin mitophagy; reduces mtROS-driven NLRP3 activation	HK-2 cells; diabetic mice	Reduced NLRP3 inflammasome activation and renal dysfunction	[57]
RU.521 (cGAS inhibitor)/genetic cGAS–STING deletion	Upstream pathway blockade	Kidney injury models (AKI/CKD)	Reduced inflammation and fibrosis	[24,48,80,123,124]
NLRP3 inhibitors (e.g., MCC950-class)	Downstream inflammasome blockade	Multiple kidney disease models	Improved renal outcomes	[125,126,127,128]

## Data Availability

The original contributions presented in this study are included in the article. Further inquiries can be directed to the corresponding author.
